# Prognostic Value of Frailty and Other Risk Factors in Patients Undergoing Lower Limb Revascularisation or Primary Major Lower Limb Amputation

**DOI:** 10.7759/cureus.111808

**Published:** 2026-06-30

**Authors:** Yang Song Wash, Walid Alnatsheh, Anthony Lee, Ayman Alshiekh, Stephen Rogers, Faris Saleh, Mohammed Ashrafi, Stephen Ball

**Affiliations:** 1 Vascular Surgery, Manchester Royal Infirmary, Manchester, GBR; 2 Vascular Surgery, Manchester University NHS Foundation Trust, Manchester, GBR

**Keywords:** amputation, clinical frailty score, major limb amputation, morbidity and mortality, revascularisation surgery

## Abstract

Objectives

Patients requiring revascularisation are increasingly becoming older, more comorbid, and deconditioned. Frailty is associated with adverse outcomes, but its role in patients undergoing revascularisation remains underexplored. The study aimed to examine the role of frailty in amputation rates, amputation-free survival and all-cause mortality.

Method

Patients aged ≥ 18years in a tertiary centre in the United Kingdom, who underwent open revascularisation, and primary amputation between September 2022 and May 2024. Electronic clinical records till January 2025 were reviewed, collated and analysed. Frailty was assessed preoperatively by anaesthetists using the Rockwood Clinical Frailty Scale (CFS), and a score was assigned

Results

Four hundred and seven patients underwent revascularisation, and 89 had primary amputation. In the revascularisation group, 55 deaths (13.5%) and 46 (11.3%) major amputations occurred. CFS ≥ 6 was present in 116 (29%) patients who underwent revascularisation and independently predicted both amputation (HR 3.13, 95% CI 1.62-6.06, p<0.001) and all-cause mortality (HR 1.27, 95% CI 1.04-1.55, p=0.017). Diabetes also increased amputation risk (HR 2.93, 95% CI 1.51-5.67, p=0.001). In the primary amputation group, mortality was associated with age and diabetes, but not with frailty. However, patients with CFS ≥ 6 died earlier (p < 0.023).

Conclusion

Frailty plays an important role in predicting outcomes. Patients with higher frailty scores were significantly more likely to experience major amputation or death, particularly within the first year after their procedure. This early postoperative period appears to be a key window, during which frail patients may benefit from closer monitoring and support.

## Introduction

Revascularisation remains a cornerstone of vascular surgery, and the demographics of patients undergoing these interventions are shifting [1]. Increasingly, vascular surgeons are managing older, more comorbid, and deconditioned patients. Age has long been recognised as a significant contributing factor to reduced physiological reserve and a higher risk of postoperative complications. However, chronological age alone does not fully explain the complexity of an individual’s vulnerability to adverse outcomes [2].

Frailty is a multifaceted syndrome marked by diminished physiological reserves and reduced resilience to stressors, which arises from cumulative decline across multiple organ systems. It is a state of increased vulnerability, where physiological insults can lead to disproportionately adverse outcomes [3].

Frail patients are often poor candidates for extensive or repeated revascularisation procedures due to their limited physiological capacity, and the range of surgical options available to them is often restricted. Interventions that might offer the best long-term outcomes in fitter individuals may pose unacceptable risks in frail patients. Surgeons may therefore be compelled to pursue less invasive or palliative strategies, which may carry reduced durability but are better tolerated in this vulnerable population [4].

While traditional risk stratification tools rely heavily on age and comorbidity indices, frailty assessments offer a more holistic understanding of patient risk [5]. Tools such as the Rockwood Clinical Frailty Scale (CFS), Fried Frailty Phenotype, and the Frailty Index have been used to assess frailty across surgical disciplines, including vascular surgery [5-8]. These instruments provide insight into a patient’s functional capacity, cognitive status, nutritional reserve, and overall resilience [5,9].

Frailty is increasingly recognised as a promising prognostic indicator for postoperative outcomes [10]. This study aimed to examine the association between frailty and different outcomes, such as major lower limb amputation rates, amputation-free survival, and all-cause mortality in patients undergoing lower limb revascularisation. It also sought to find other key predictors of these outcomes.

## Materials and methods

Study design

This was a single-centre retrospective observational study of patients who had lower limb revascularisation procedures and primary major lower limb amputation. The hospital electronic theatre records were used to identify all patients who had these procedures between September 2022 and May 2024, and followed up for outcomes to January 2025. Approval of the audit of patient case notes requested and received from the institution.

Study setting

This study was carried out in the Vascular Surgery department of Manchester Royal Infirmary, United Kingdom.

Patient selection and outcomes

Data of patients aged 18 years and older who underwent either revascularisation procedures or primary major amputation were collated.

The revascularisation group comprised patients treated either electively or as emergencies. Procedures included femoral endarterectomy (with or without intraoperative angiography), bypass surgeries (aortobifemoral, axillofemoral, and infrainguinal), and embolectomies.

The primary amputation group included patients who underwent major limb amputation (defined as all amputations above the ankle) without any preceding attempt at revascularisation.

The outcome considered was the determination of predictive factors for events (major limb amputation, amputation-free survival, and all-cause mortality) among patients who had revascularisation and primary amputation. Adverse events in this study refer to major limb amputation and or all-cause mortality, and days to amputation and days to mortality in patients who had these events were recorded.

Data sources

The electronic clinical records of included patients were reviewed to collate study outcomes. Data were stored in password-protected Microsoft Excel for Microsoft 365 spreadsheets, version 2406 (Microsoft Corporation, Redmond, WA, USA), on the secure intranet of Manchester University NHS Foundation Trust, with access restricted to members of the study team. The Rockwood CFS was assessed by anaesthetists preoperatively, unaware of the study, and a score was assigned [11]. Follow-up ranged from seven to 28 months, with a median follow-up of 15 months.

Data analysis

Parametric and non-parametric tests were used to analyse continuous and non-continuous variables as appropriate. Univariate Cox proportional hazards regression was performed considering days to event for different variables, using a significance threshold of p < 0.1 to identify potential predictors. Variables meeting this threshold were then included in a stepwise multivariable Cox regression analysis, with statistical significance in the final model defined as p < 0.05. Regression analyses were done using the analytical software R (R core team 2024) [12]. Results are reported in line with Strengthening the Reporting of Observational Studies (STROBES) format [13].

## Results

Four hundred and seven patients underwent revascularisation procedures during the study period, and 89 patients had primary amputation.

In patients who had revascularisation, the median age was 68 years, with a mean of 68.1 ± 10.3 years. Of these, 297 patients (73%) were male, resulting in a male-to-female ratio of approximately 2.7:1. More demographic information and distribution of risk factors are seen in Table 1. In 407 revascularisation procedures, 53 (13.0%) underwent common femoral artery endarterectomy (CFAE) without angiography, 165 (40.5%) underwent bypass surgery (prosthetic and vein bypasses), and 183 patients (45.0%) received hybrid procedures, defined as CFAE or bypass with angiography ± stenting. There was no statistically significant association between procedure type and either major amputation or mortality.

**Table 1:** Showing distribution of risk factors and outcome in patients who had revascularisation. N (%) = number (percentage) for categorical variables; median (IQR) = median (interquartile range) for continuous variables. Frailty score, reached after assessment of frailty using the Rockwood Clinical Frailty Scale, a free tool used to assess frailty [6]. IQR, interquartile range; COPD, chronic obstructive pulmonary disease.

Variables	Total (N (%)/median(IQR))	No amputation or death (N (%)/median (IQR))	Amputation only (N (%)/median (IQR))	Death only (N (%)/median (IQR))	Amputation and death (N (%)/median (IQR))
Age, median (IQR)	68 (61–76)	68 (61–75)	68 (60–74)	74 (68–79)	76 (73–81)
Frailty score, median	4 (3–6)	4 (3–5)	6 (4–6)	5 (4–6)	6 (4–6)
High frailty (CFS ≥ 6)	116 (29%)	70 (22%)	20 (57%)	19 (43%)	7 (64%)
Diabetes mellitus	191 (47%)	132 (42%)	27 (77%)	25 (57%)	7 (64%)
Hypertension	288 (71%)	225 (71%)	26 (74%)	29 (66%)	8 (73%)
Ischaemic heart disease	135 (33%)	94 (30%)	15 (43%)	23 (52%)	3 (27%)
COPD	144 (35%)	114 (36%)	8 (23%)	17 (39%)	7 (45%)
Current/former smoker	190 (47%)	141 (44%)	18 (51%)	24 (55%)	7 (64%)

During the follow-up period, 55 deaths (13.5%) occurred. The all-cause mortality rates were 2.9% at 30 days, 5.2% at 90 days, and 11.5% at 1 year. The results of the univariate and multivariate analyses examining risk factors for mortality are summarised in Appendix 1.

Among patients who underwent initial revascularisation, 46 patients (11.3%) subsequently required major limb amputation during the follow-up period. The predictors of major amputation are presented in Appendix 2. The cumulative amputation rates were 2.2% at 30 days, 6.4% at 90 days, and 9.6% at 1 year, as detailed in Appendix 3.

Frailty was strongly associated with adverse outcomes. Among the 116 patients with a CFS ≥6, 46 patients (40%) experienced adverse events. In comparison, 44 patients (15%) of the 291 patients with a CFS <6 experienced similar events.

In addition, 89 patients underwent primary major amputation without an attempt at revascularisation. The Cox regression analysis evaluating risk factors for mortality following primary amputation is presented in Appendix 4.

## Discussion

**Figure 1:**
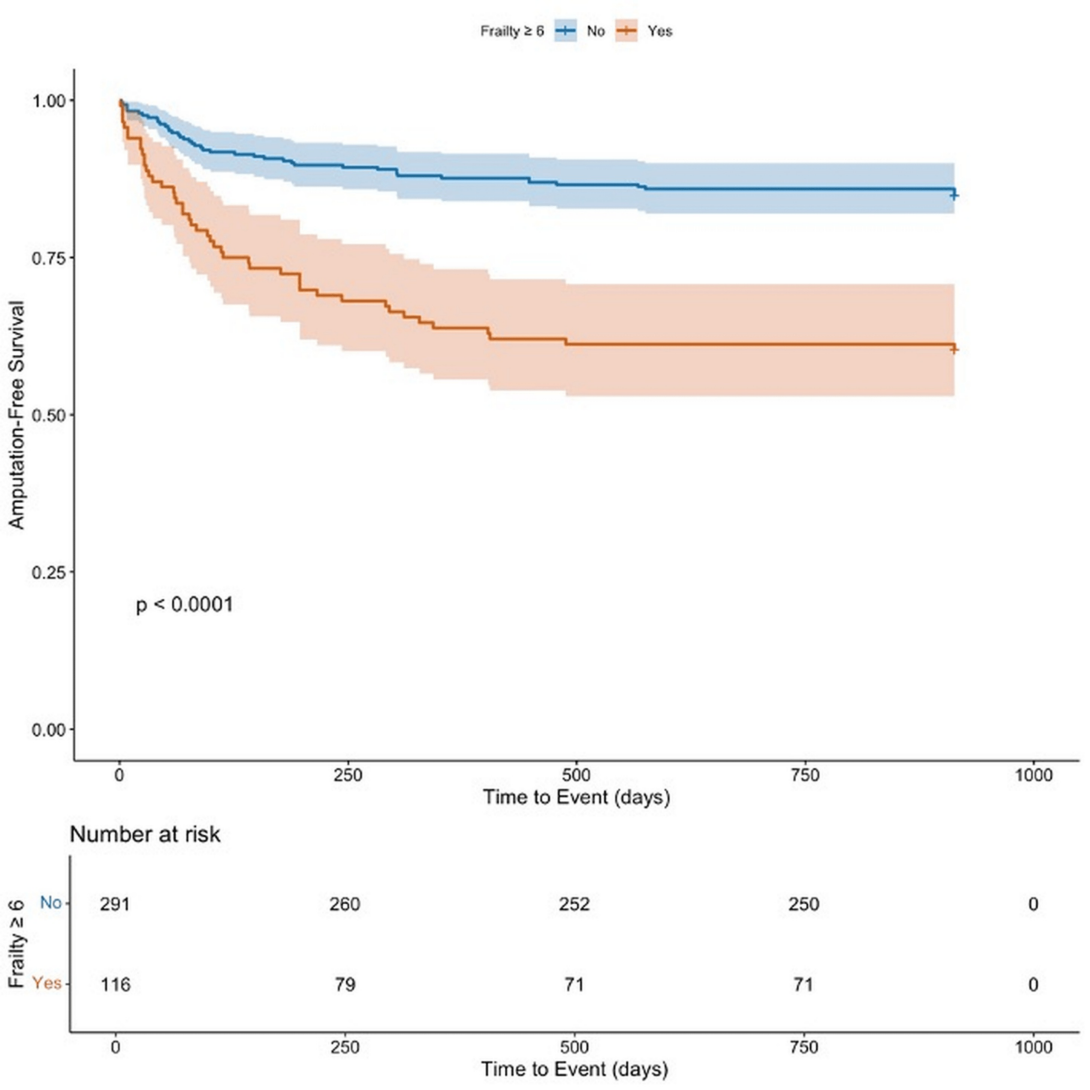
Kaplan-Meier curve showing amputation free survival by frailty group after open revascularisation. Frailty score, as assessed by the Rockwood Clinical Frailty Scale [6].

This study reinforces the growing recognition of frailty as a powerful predictor of adverse outcomes following lower limb revascularisation [10]. Forty-six (40%) of patients with a CFS ≥ 6 experienced adverse events compared to 44 (15%) in those with CFS less than 6. These findings are consistent with previous work demonstrated by Takeji et al., and further supported by Zhang et al. [14,15]. The survival curve by frailty group (Figure 1) diverges early and continues to separate, suggesting that frailty is an early and persistent risk factor for adverse events after revascularisation.

**Figure 2:**
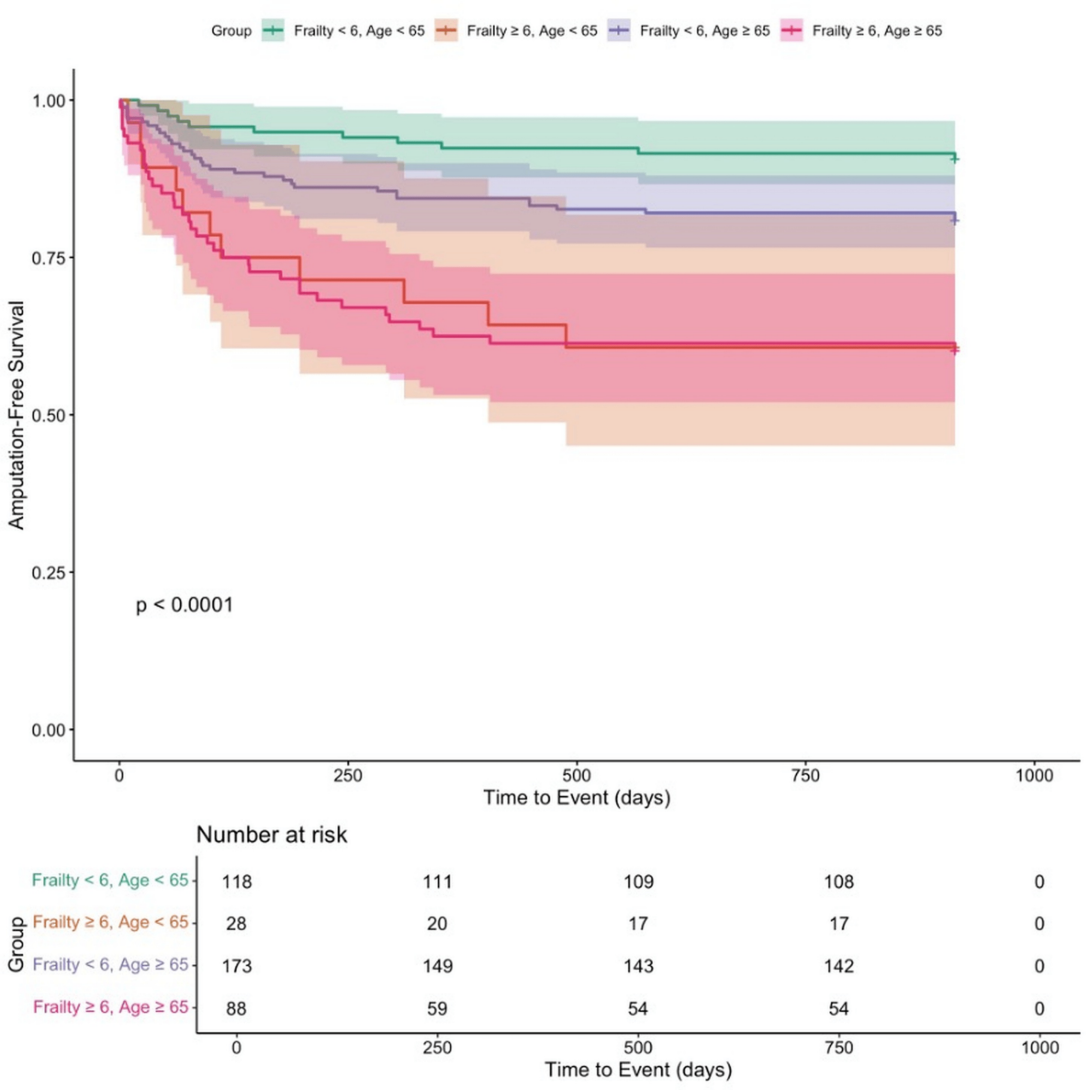
Kaplan-Meier curves showing amputation free survival by age and frailty after open revascularisation. Frailty score, as assessed by the Rockwood Clinical Frailty Scale [6].

Increasing age, as expected, was found to have a significant association with mortality. However, as illustrated in the Kaplan-Meier curve depicting the impact of age and frailty on amputation-free survival in patients undergoing revascularisation (Figure 2), the worst outcomes occurred in patients with CFS ≥ 6, regardless of their age group. Both younger and older patients with CFS ≥ 6 experienced early and sustained poor outcomes, lasting up to approximately one year. The impact of frailty was seen even in younger patients with CFS ≥ 6, who showed a steady decline over time. The lowest adverse event rate was in the younger patients with CFS < 6.

Diabetes and advancing age are well-established risk factors for postoperative complications. In the present study, diabetes was associated with an almost threefold increased risk of amputation on multivariate regression analysis (HR 2.93, 95% CI 1.51-5.67, p=0.001), but it was not an independent predictor of mortality, as the association did not reach statistical significance. This shows the role of diabetes in shaping postoperative limb-related outcomes, but on its own does not predict mortality significantly [16].

The overall mortality and amputation rate during the follow-up period were 13.5% and 11.3%, respectively, with the associated risk factors presented in Appendix 1 and Appendix 2. The Kaplan-Meier curve in Figure 2 demonstrates that the majority of adverse events occurred within the first 12 months following revascularisation. Event rates then declined sharply, reaching a plateau around the 13 to 24-month mark. Notably, only 0.5% of amputations and 1.8% of deaths occurred between one and two years post-procedure. This pattern shows that the first postoperative year is a critical window, during which patients, particularly those who are frail, are most vulnerable and could benefit more from focusing healthcare resources and multidisciplinary support during this phase to improve outcomes [17].

Frailty demonstrated a stronger and more consistent association with adverse outcomes than traditional cardiovascular risk factors. As seen in the tables showing risk factors for mortality and amputation, frailty is seen to increase amputation risk threefold (HR 3.13 (95% CI 1.62-6.06), p < 0.001) and mortality risk by 27% (HR 1.27 (95% CI 1.04-1.55), p = 0.017), agreeing with work by Vaes et al. [18,19]. In contrast, comorbidities such as COPD, hypertension, smoking, and ischaemic heart disease did not consistently predict adverse outcomes. While ischaemic heart disease (IHD) reached statistical significance in univariate analysis, its association with outcomes was no longer significant after adjustment for frailty and age in multivariate models. These findings suggest that frailty reflects physiological vulnerability beyond what is explained by traditional cardiovascular risk factors [20,21].

These findings buttress the perspective that frailty, as a marker of global physiological reserve and systemic vulnerability, provides superior prognostic value compared to evaluating isolated organ-specific conditions. Incorporating frailty in prediction models may offer a more accurate and individualised approach to risk assessment, guiding both clinical decisions and discussions with patients and families [22]. With the right prediction tools, clinicians are able to weigh the benefits of technical success against the significant risk of complications more precisely, particularly during the critical period between 90 days and one year, when adverse events are most likely to occur [23,24]; as this would influence the choice of intervention, if offered at all.

**Figure 3:**
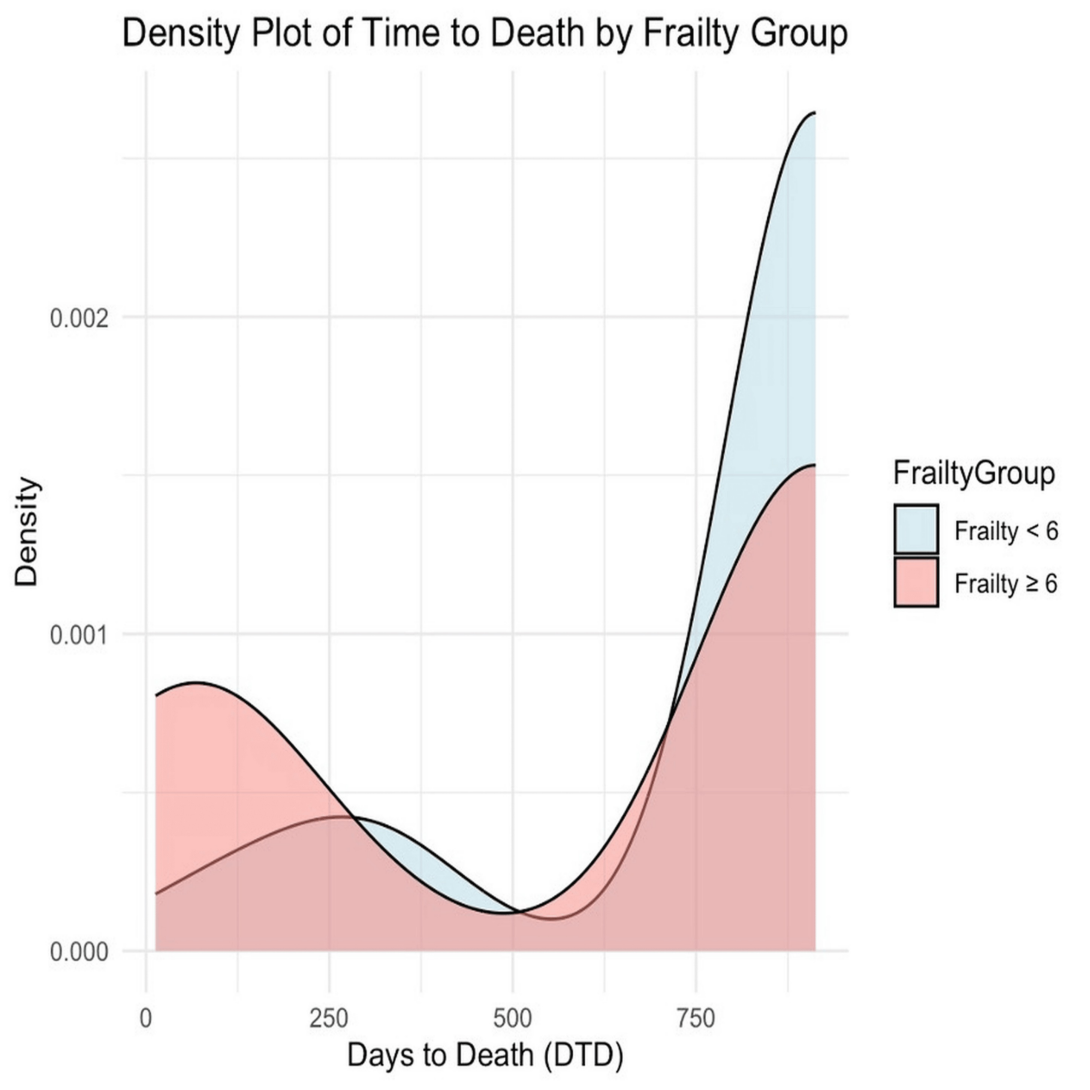
Density plot of distribution of time to death by frailty score in patients who had primary amputation (Mann-Whitney U test p < 0.023). Frailty score, as assessed by the Rockwood Clinical Frailty Scale [6].

Patients who underwent primary amputation had similar frailty scores (median and mean) among survivors and non-survivors, with no significant difference found after regression analysis. This similarity may be explained by surgeons opting for primary amputation in patients deemed too frail or high-risk for revascularisation. However, a significant difference was seen in time to death among patients who had primary amputation, as shown by the density plot in Figure 3 and confirmed by the Mann-Whitney test (P < 0.023), indicating that patients with frailty scores of 6 or higher experienced earlier peaks in mortality [25].

**Figure 4:**
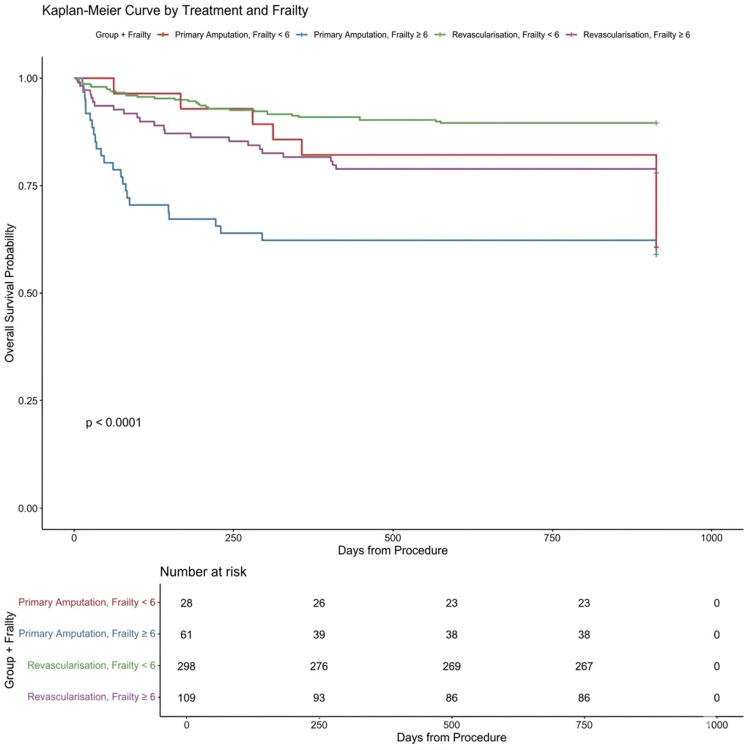
Kaplan-Meier curve showing survival probability for treatment type and frailty with time. Frailty score, as assessed by the Rockwood Clinical Frailty Scale [6].

From the study, increasing frailty is associated with worse outcomes, regardless of the treatment approach, as seen in Figure 4. The worst outcomes were observed in patients with a frailty score of 6 or higher who underwent primary amputation. In contrast, patients with similar frailty scores who had revascularisation demonstrated a better survival advantage. Among patients with a frailty score below 6, survival rates were initially comparable across treatment groups for about a year. After this point, survival declined even more in patients who had primary amputation, while survival in patients who underwent revascularisation held steady after this point, reflecting that the risk of mortality for patients who undergo revascularisation is highest in the perioperative period [26], and when a patient survives this period, the risk of mortality in this group reduces significantly.

Limitations of the study

This study was not randomised, which may affect the representativeness of the sample. However, all patients who underwent revascularisation during the study period were included, regardless of whether the procedure was performed electively or in an emergency setting, suggesting a real-world reflection. However, as a retrospective study, it may be subject to inherent biases, but efforts were made to include all eligible patients who met the inclusion criteria, with follow-up conducted for all relevant outcomes. Additionally, the subjective nature of the CFS is an important consideration. However, frailty assessments were typically performed by a senior clinician; usually, the anaesthetist responsible for the case. Importantly, they were not aware of the study, thereby reducing the potential for observer bias.

## Conclusions

This study shows that frailty plays an important predictive role for outcomes in patients undergoing revascularisation or primary amputation, particularly within the first year after procedure. This early postoperative period appears to be a key window, during which frail patients may benefit from closer monitoring and support. Routine assessment of frailty could help clinicians make better-informed decisions and improve outcomes in this high-risk population.

## Appendices

Appendix 1

**Table 2:** Cox regression analysis of risk factors for mortality after open revascularisation. CFS, Clinical Frailty Scale; DM, diabetes mellitus; HTN, hypertension; IHD, ischaemic heart disease; COPD, chronic obstructive pulmonary disease.

Variable	Univariate hazard ratio	Univariate p-value	Univariate 95% confidence interval	Multivariate hazard ratio	Multivariate p value	Multivariate 95% confidence interval
Age	1.07	<0.001	1.04 – 1.10	1.06	<0.001	1.03 – 1.10
CFS ≥ 6	2.26	0.003	1.32 – 3.84	1.27	0.017	1.04 – 1.55
DM	1.65	0.068	0.96 – 2.81	1.60	0.089	0.93 – 2.77
HTN	0.82	0.487	0.47 – 1.44	–	–	–
IHD	1.86	0.021	1.10 – 3.16	1.59	0.091	0.93 – 2.71
COPD	1.23	0.462	0.72 – 2.10	–	–	–
Smoking	1.32	0.306	0.77 – 2.26	–	–	–

Appendix 2

**Table 3:** Cox regression analysis of risk factors for amputation after open revascularisation. CFS, Rockwood Clinical Frailty Scale; DM, diabetes mellitus; HTN, hypertension; IHD, ischaemic heart disease; COPD, chronic obstructive pulmonary disease.

Variable	Univariate hazard ratio	Univariate p-value	Univariate 95% confidence interval	Multivariate hazard ratio	Multivariate p-value	Multivariate 95% confidence interval
Age	1.015	0.315	0.99 – 1.05	–	–	–
CFS ≥ 6	3.546	<0.001	1.99 – 6.34	3.13	<0.001	1.62 – 6.06
DM	3.397	<0.001	1.76 – 6.56	2.926	0.001	1.51 – 5.67
HTN	1.149	0.680	0.60 – 2.22	–	–	–
IHD	1.404	0.203	0.83 – 2.37	–	–	–
COPD	1.222	0.501	0.69 – 2.18	–	–	–
Smoking	1.428	0.238	0.79 – 2.58	–	–	–

Appendix 3

**Table 4:** Amputation-free survival and death by frailty group N (%), number (percentage); AFS, amputation-free survival; CFS: Rockwood Clinical Frailty Scale.

Frailty group	AFS 30d N (%)	AFS 90d N (%)	AFS 1yr N (%)	Death 30d N (%)	Death 90d N (%)	Death 1yr N (%)
CFS < 6	285 (98%)	286 (98%)	262 (90%)	6 (2%)	5 (2%)	29 (10%)
CFS ≥ 6	108 (93%)	112 (97%)	90 (78%)	8 (7%)	4 (3%)	26 (22%)

Appendix 4

**Table 5:** Cox univariate regression showing risk factors for mortality after primary amputation. N (%), number (percentage) for categorical variables; median (IQR), median (interquartile range) for continuous variables; CFS, Rockwood Clinical Frailty Scale; DM, diabetes mellitus; HTN, hypertension; IHD, ischaemic heart disease; COPD, chronic obstructive pulmonary disease.

Risk factor	Survived (N (%)/median (IQR))	Died (N (%)/median (IQR))	P-value	Hazard ratio
Age	64.0 (54.0–73.0)	77.0 (64.8–80.2)	0.0010	1.05
DM	27 (50.9%)	27 (75.0%)	0.0395	2.89
HTN	35 (66.0%)	26 (72.2%)	0.7009	1.34
COPD	16 (30.2%)	16 (44.4%)	0.2500	1.84
IHD	19 (35.8%)	20 (55.6%)	0.1049	2.24
CFS ≥ 6	36 (67.9%)	25 (69.4%)	0.430	1.07
